# Supplementary material for: Genome and Transcriptome Identification of a Rice Germplasm with High Cadmium Uptake and Translocation
Source: Plants (Basel). 2023 Mar 8;12(6):1226. doi: 10.3390/plants12061226 (PMC10056766; doi:10.3390/plants12061226)
Supplement: Supplementary file 1 [file plants-12-01226-s001.zip › plants-2266590-supplementary.pdf]

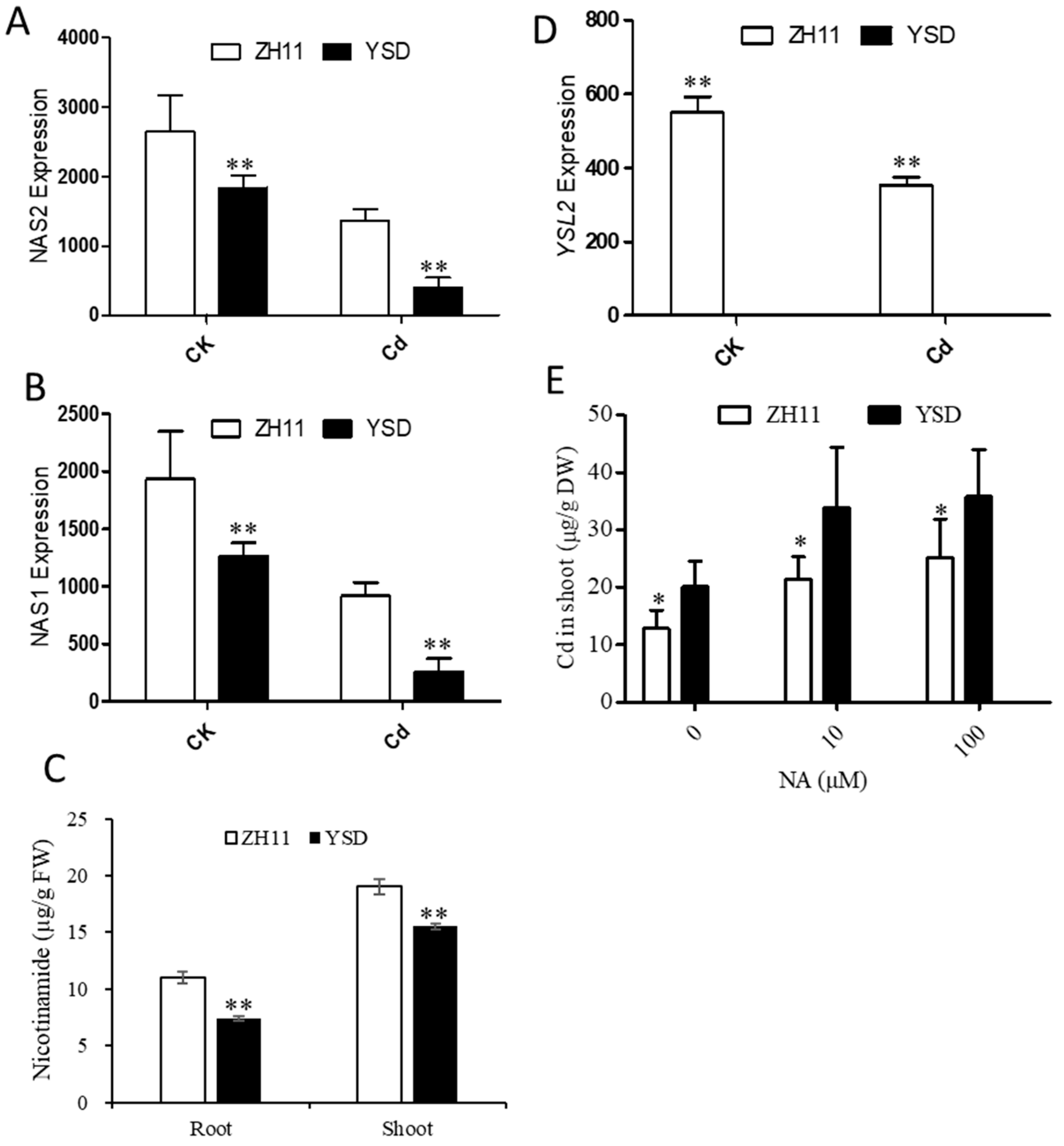

Supplementary Figure S1. Nicotinamide treatment promoted the translocation of cadmium in rice (A-D) After 4 weeks of hydroponics, rice seedlings were treated with 10 μ M Cd for 3 days, Nicotinamide synthesis gene expression level (A-B), nicotinamide content (C), and expression level of YSL2 genes were determined. Data are means (±SD), n=3. (E) 4-weeks-old hydroponics rice sedlings were treated with 10 μM Cd, 10 μM Cd plus 10μM nicotinamide and 10 μM Cd plus 100 μM for 3 days, and the Cd content in shoots was determined by ICP-MS. Data are means (±SD), n=5. Significant differences were determined using Student's t-test: *$P<0.05$; **$P<0.01$

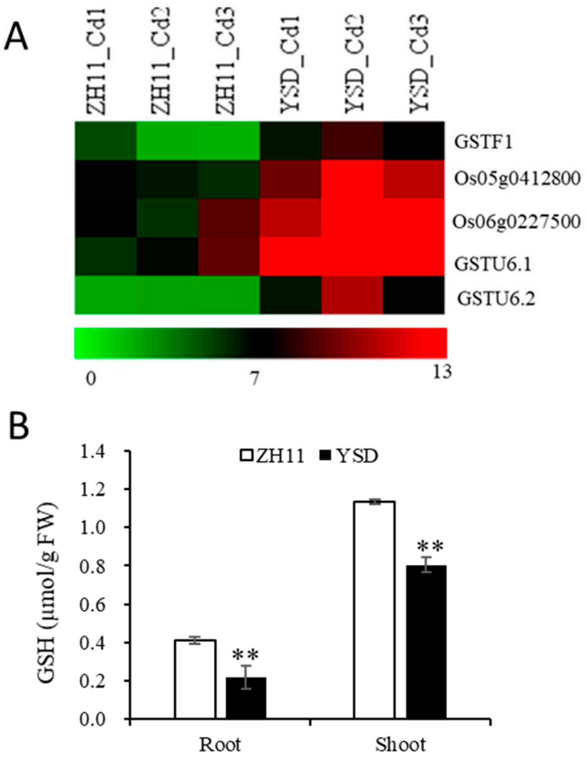

Supplementary Figure S2. GST transferase expression level and GSH content in YSD and ZH11 Rice seedling was hydroponic for 4 weeks and treated with 10 µM cadmium Cd for 3 days. (A) Differentially expressed genes (DEGs) involved in GST transferase between YSD and ZH11 root. (B) GSH content in YSD and ZH11 root and shoot. Data are means (±SD), n=3. Significant differences were determined using Student's t-test: **P*<0.05; ***P*<0.01

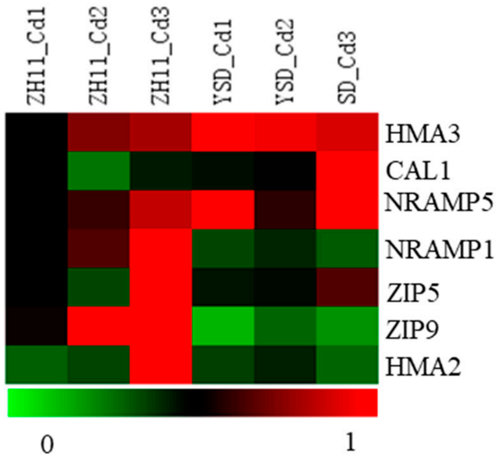

Supplementary Figure S3. Expression level of Cd uptake and translocation gene betweenYSD and ZH11 root under Cd treatments. Transcriptional profiling of gene responsible for Cd uptake (NRAMP5/ NRAMP1/ZIP5/ZIP9), translocation (HMA2/CAL1) and vacuolar sequestration (HMA3).
